# Editorial: Effects of medicinal homologous foods on immunity through intestinal flora

**DOI:** 10.3389/fimmu.2023.1358254

**Published:** 2024-01-04

**Authors:** Zhoujin Tan, Kangxiao Guo, Xihong Zhou, Xinhua Shu

**Affiliations:** ^1^ College of Chinese Medicine, Hunan University of Chinese Medicine, Changsha, Hunan, China; ^2^ Department of Pharmacy, Changsha Health Vocational College, Changsha, Hunan, China; ^3^ Institute of Subtropical Agriculture, Chinese Academy of Sciences, Changsha, Hunan, China; ^4^ Department of Biological and Biomedical Sciences, Glasgow Caledonian University, Glasgow, United Kingdom; ^5^ Department of Vision Science, Glasgow Caledonian University, Glasgow, United Kingdom

**Keywords:** medicinal homologous food, gut microbiota, metabolic disease, oxidative stress, inflammation

The human gastrointestinal tract harbours a complex community of microorganisms, called gut microbiota, that includes bacteria, fungi, virus, archaea and protozoa (1). Bacteria predominate in human gut microbiota, comprising over 2000 species and 9.9 million microbial genes (1). There are 50 bacterial phyla identified in gut microbiota, of which *Firmicutes* and *Bacteroidetes* are most abundant, accounting for approximately 90% of total microbiota (2). Gut microbiota develops dynamically from birth to childhood, becoming relative stable during adulthood. Many factors, such as host genetics, geographical location and antibiotic treatment, regulate the composition of gut microbiota. Diet is considered as a predominant influence on gut microbiota: mice fed with a high-fat-diet (HFD) undergo a composition change of gut microbiota after 24hours. Gut microbiota has co-evolved with humans over thousands of years and forged a mutually beneficial relationship. It has been shown to have multiple functions in the maintenance of the intestinal mucosal barrier, digestion of complex nutrients such as dietary fibres and synthesis of metabolites (e.g. short-chain fatty acids - SCFAs), inhibition of growth of pathogenic bacterial species, regulation of intestinal and systemic immune homeostasis, modulation of intestinal and systemic hormonal responses, and elimination of exogenous drugs/toxins. Dysbiosis, characterized by an imbalance of microbial composition and a decrease in microbial diversity of gut microbiota, is associated with various types of diseases, including metabolic, neurodegenerative and mental disorders (1, 2).

The term ‘medicinal homologous foods’ (MHF) refers to a group of natural products, particularly herbal materials, that can be consumed as foods and as medicine when treating human diseases. Numerous MHF have been documented in ancient medicinal books and used over thousands of years in China. MHF have demonstrated capacity against oxidative stress, inflammation and metabolic problems. Due to multiple ingredients in MHF, it is surmised that the beneficial effects of MHF involve complicated molecular mechanisms. Recently, many studies have shown that MHF regulate gut microbiota homeostasis. In this Research Topic, we aim to collect recent progress in understanding the functional link between MHF and gut microbiota and share this updated research with the scientific community. This Research Topic encompasses four review papers and three research papers.

Obesity is defined as a body mass index ≥30 and affects approximately 770 million adults globally (https://www.worldobesity.org/about/about-obesity/prevalence-of-obesity). The significant increase in prevalence of obesity in the last three decades is primarily associated with lifestyle trends, particularly the increased intake of high-fat fast foods. A fat-enriched diet can alter the composition of gut microbiota and its transcriptome (3). Obesity is a major risk factor for metabolic disorders, such as type 2 diabetes. Li et al. investigated the therapeutic effect of walnut peptide (WP), a small molecule isolated from oil-extracted walnut meal, and found that WP treatment decreased body weight, reduced liver lesion and adipocyte volume, suppressed inflammation, and repaired the intestinal mucosal barrier in HFD fed mice. The authors also reported that WT treatment modulated the diversity and structure of intestinal microbiota and regulated the production of microbial metabolites such as SCFAs. Su et al. discussed the beneficial effects of *Astragali Radix*, a herb used prevalently in traditional Chinese medicine (TCM), against type 2 diabetes. *Astragali Radix* can regulate the abundance of SCFA-producing and inflammation-associated bacteria in mice with cisplatin-induced liver injury. In type 2 diabetic mice, *Astragali Radix* can control body weight and blood glucose via improvement of the abundance and diversity of gut microbiota. Various *Astragali Radix*-containing TCM formulas also show capacity against type 2 diabetes via the reversal of intestinal dysbiosis and the regulation of microbial metabolite production. These protective effects are further replicated in the treatment of type 2 diabetes with active compounds of *Astragali Radix*. Obesity is also associated with age-related macular degeneration (AMD), a metabolic retinal disorder that currently affects over 200 million individuals globally. Previous studies have shown that the pathogenesis and progress of AMD are connected with diet and gut microbiota (4). Cao et al. examined the therapeutic potential of Qijudihuang pill (QP), a widely used TCM, for AMD. The authors found that QP treatment decreased local and systemic lipid levels, increased antioxidant activities and inhibited inflammation in mice fed with HFD. QP treatment also reversed major HFD-induced changes in abundance, diversity, structure and metabolic pathways of gut microbiota, returning them to characteristics of control mice fed with a normal diet.

Inflammatory bowel disease (IBD) is a complex immune intestinal disorder, associated with environmental and genetic risk factors (5). Animal models have demonstrated that gut microbiota is essential for the development of IBD. IBD patients have decreased biodiversity and altered abundance of some phyla/classes/species, resulting in loss of intestinal barrier integrity, inflammation, inappropriate immune response and altered microbial metabolic pathways, which contribute to IBD pathogenesis (5). *Houttuynia cordata thumb* (HC), a member of the Saururaceae family, has been widely used in TCM to treat inflammatory disorders. Wang et al. discussed the therapeutic effects against IBD by HC ingredients and HC-based TCM formula. The authors gave examples that essential oils, polysaccharides and flavonoids from HC alleviated IBD symptoms via regulating gut microbiota, modulating immune response and maintaining intestinal mucosal barrier integrity.

This Research Topic also collected papers covering mediation of MHF ingredients in immune-associated disorders. Hu et al. reviewed literature of functional properties and underlying mechanism of curcumin, a predominant active compound of *Curcua longa* rhizome. The authors demonstrated curcumin functions against oxidative stress, inflammation and apoptosis via regulating the NRF2, NF-κB, PI3K and JAK pathways. Wang and Hu also reviewed literature about the protective effects of polyphenols against osteoporosis, a common metabolic bone disorder. Dysregulation of gut microbiota is associated with increase inflammation and bone resorption, contributing to formation and progression of osteoporosis. Polyphenols can enhance bone formation, alleviate inflammation and treat osteoporosis possibly in part by regulating gut microbiota homeostasis and bacterial metabolite production. Zuurveld et al. investigated the effects of milk oligosaccharides (2’-fucosyllactose, 2’FL and 3-fucosyllactos, 3FL) in suppression of ovalbumin-induced allergy *in vitro* and *in vivo*. The authors demonstrated that 2’FL and 3FL differentially modulated ovalbumin-induced immune activation *in vitro*, which functionally collaborated with butyrate. 2’FL or 3FL-containing diet altered the serum immunoglobulin levels and the T cell populations in mesenteric lymph nodes and increased production of cecum SCFAs in ovalbumin-allergic mice, when compared to untreated controls.

In summary, MHF have shown beneficial effects in prevention and treatment of various disorders in humans, involving multiple functional compounds and signalling pathways. This Research Topic collection provides a platform for exchange of recent developments in this area and facilitates further development of new treatment with MHF for a wide range of human diseases.

## Author contributions

ZT: Writing – review & editing. KG: Writing – review & editing. XZ: Writing – review & editing. XS: Writing – review & editing, Writing – original draft.
